# The critical role of diet, exercise, and sleep in shaping the gut microbiota of children with idiopathic short stature: a Retrospective study

**DOI:** 10.3389/fimmu.2025.1566722

**Published:** 2025-08-01

**Authors:** Qin Zeng, Jun Zhu, Yanling Hu, Shaoyu Su, Jing Chen

**Affiliations:** ^1^ Department of Pediatrics Nursing, West China Second University Hospital, Sichuan University, Chengdu, China; ^2^ Key Laboratory of Birth Defects and Related Diseases of Women and Children (Sichuan University), Ministry of Education, Chengdu, Sichuan, China; ^3^ National Office for Maternal and Child Health Surveillance of China, National Center for Birth Defect Surveillance of China, Department of Pediatrics, West China Second University Hospital, Chengdu, China

**Keywords:** idiopathic short stature, intestinal microbiota, lifestyle factors, correlation analysis, 16S rRNA gene sequencing

## Abstract

**Objectives:**

To investigate the gut microbiota in children with varying degrees of idiopathic short stature (ISS) and to examine the relationship between their intestinal microbiota and lifestyle factors, including diet, exercise, medication, sleep, and psychological state.

**Methods:**

A retrospective study involving 58 ISS children was conducted from May to October 2022. Lifestyle data were collected using questionnaires. Fecal samples were analyzed using 16S rRNA gene sequencing to assess microbiota diversity and composition. LEfSe analysis identified differential bacterial communities between ISS-2SD and ISS-3SD groups (LDA score ≥ 2.5). Spearman correlation analysis explored the relationship between microbiota diversity, dominant taxa, and lifestyle factors, visualized in a heatmap.

**Results:**

No significant differences in alpha diversity of intestinal microbiota were observed among children with varying degrees of short stature at the ISS, but differences were noted in the dominant microbiota. The ISS-2SD group had *Leptotrichiaceae* and *Sneathia* as predominant members, whereas the ISS-3SD group was dominated by *Lachnoclostridium*, *Thermous_scotoductus* and *Thermoles.* Correlation analysis revealed that microbiota diversity was linked to diet, especially legume consumption (Shannon index: r=0.372, *P*=0.004; Simpson index: r=0.379, *P*=0.003). At the genus level, *Prevotella* was positively correlated with beverage intake (r=0.262, *P*=0.047) and sleep quality (r=0.324, *P*=0.013), while *Ezakiella* was negatively correlated with meat intake (r=-0.297, *P*=0.024), Other genera exhibited significant correlations with diet, exercise, and sleep.

**Conclusion:**

Children with varying degrees of short stature exhibited differences in their dominant intestinal microbiota. Diet, exercise, and sleep appear to be significant factors influencing these microbial changes in ISS children.

## 1 Introduction

ISS refers to the short stature with normal growth hormone (GH) level and no underlying pathological state. Although living in similar environment, the height is 2 standard deviations (-2SD) lower than the average value of children of the same age, gender and race (1). The physical and social harm caused by short stature for children is very obvious. Research has indicated that ISS can engender substantial psychological and social ramifications for afflicted pediatric populations, encompassing an augmented propensity for anxiety, depressive disorders, and social maladjustment (2). Children exhibiting short stature frequently encounter underestimation from their peer groups, educators, and familial caregivers, often enduring instances of derision, social exclusion, or overprotective behaviors, potentially predisposing them to social isolation and discriminatory practices (3). The aforementioned physical characteristic renders these children more vulnerable to emotional adversities, including heightened anxiety and depressive symptomatology. Moreover, extant research underscores that children with ISS may confront distinct emotional and behavioral challenges, including instances of bullying, social isolation, and perceived stigmatization, all of which may contribute to the manifestation of internalizing behaviors, such as elevated anxiety and depressive tendencies (2, 4). In addition, being short in stature can also affect an individual’s career development, such as restrictions on further education, career choices, and joining the military, and may also affect their choice of spouse in adulthood (5).

Several studies have shown that gut microbiota may be an important factor in the development and outcome of ISS (6, 7). Notably, Lazar et al. (8)and Li et al. (9) have made significant contributions to the understanding of the microbiome’s relationship with ISS, underscoring the potential involvement of gut microbiota in the pathophysiology of ISS. Furthermore, existing studies have found that the gut microbiota is related to individual internal and external factors, including birth, diet, living environment, host genetics, age, drug intake, and disease (10–12). For example, Surono et al. (13) observed that the gut microbiota in children with developmental delay may be associated with factors such as gender, height, body mass index (BMI), sampling location, place of origin and residence, socioeconomic and hygiene status, dietary habits, presence of anorexia nervosa, and age of onset, the specific hospital or clinic where the fecal samples were collected (sampling location), place of origin and residence, family economic and hygiene status, dietary status, presence of anorexia nervosa, and the age at which the children were first diagnosed with developmental delay (age of onset). Existing studies have found that the gut microbiota in children with nutritional short stature is closely associated with factors such as age, dietary habits, medication use, physical activity, sleep patterns, bowel regularity, and psychological well-being (14). Nutritional short stature is typically caused by inadequate nutrition, leading to growth retardation, and is often linked to malnutrition, poor dietary intake, and recurrent infections, thereby affecting children’s growth and development (14). Wang et al. (15) demonstrated that Bacteroides fragilis polysaccharide A (PSA) can modulate the gut microbiota and ameliorate aberrant voriconazole metabolism via the inhibition of the TLR4/NF-κB pathway. Additionally, Wang et al. (16) reported that Astragalus polysaccharide (APS) enhances voriconazole metabolism under inflammatory conditions by regulating the gut microbiota. These findings highlight the potential of gut microbiota in modulating drug metabolism and its implications for pediatric health. Collectively, the daily lifestyle behaviors influencing alterations in gut microbiota in children with dwarfism primarily encompass five categories: diet, exercise, sleep, medication, and psychological factors.

Previous studies have have established the critical influence of the gut microbiota on pediatric development and overall health. Nevertheless, a conspicuous lacuna exists within the extant literature concerning the alterations in gut microbiota and associated parameters among children diagnosed with ISS, stratified by the severity of their growth deficiency. This deficiency is particularly pronounced both nationally and globally. To address this critical knowledge gap, our research was specifically directed towards ISS-afflicted children residing in southwestern China, an area characterized by a high incidence of ISS cases. We strategically selected ISS-diagnosed children with the highest frequency of ISS-related clinical encounters at our study site as the subjects for this investigation. Employing 16S rRNA gene sequencing technology, our objective was to elucidate the modifications in gut microbiota composition and related factors among ISS-diagnosed children exhibiting varying degrees of short stature within this specific geographical context.

Additionally, we would like to highlight that the gut microbiota differences between ISS children and healthy controls have been extensively discussed in our previously published study (17). This study demonstrated significant dysbiosis in the ISS group compared to healthy controls, particularly with an enrichment of pro-inflammatory species, thereby providing a robust background for the current investigation on microbiota variation within ISS subgroups.

Through this experiment, we aim to answer the following questions: Are there differences in gut microbiota among ISS children with different levels of dwarfism? Is there a correlation between changes in gut microbiota and lifestyle behaviors in ISS children? This research aims to identify new targets for regulating gut microbiota through probiotics and health science-based lifestyle behaviors, as well as for ISS prevention, etiological treatment, and precision medical care.

## 2 Materials and methods

### 2.1 Research participants

Fifty-eight children diagnosed with ISS following growth hormone stimulation tests at a specialized women’s and children’s hospital in Sichuan Province from May 2022 and October 2022. These children are residents of Chengdu, Han ethnicity, and have a balanced gender. They meet the ISS diagnostic criteria: height below -2SD or the 3rd percentile of the average height of children of the same gender, age, and race, but normal at birth and infancy, no underlying pathological conditions causing dwarfism, normal GH stimulation test, and no genetic metabolic or chronic diseases. The diagnostic criteria used here are based on the guidelines outlined by Pedicelli et al. (1) in their discussion of ISS definition and treatment. The inclusion criteria include height consistent with ISS diagnosis, age 6–12 years old, no organic, genetic, malformation, or chronic diseases, no gastrointestinal inflammation or examination within the past 3 months, and children and parents’ consent to participate in the study. Exclusion criteria encompass precocious puberty, isolated early breast development, usage of antibiotics or probiotics within the previous three months, psychological or severe emotional disorders. Additional exclusion criteria involve non-cooperation during inspection and sample collection, failure in specimen testing, withdrawal from the study before completion, and absence of more than 20% of the primary observation indicators.

### 2.2 Research tool

This study used two questionnaires, the “General Information Survey Form” and the “Questionnaire on Factors Related to Intestinal microbiota”. The former covers information on children, family, and pregnancy, while the latter pertains to diet, exercise, medication, sleep, and psychological status.

The dietary questionnaire was developed based on the Nutrition Guidelines and Food Frequency Questionnaire (FFQ) (18, 19), including the average intake and frequency of staple foods (rice, noodles, steamed buns, and a total of eight items), meat (pork, beef, mutton, and a total of nine items), dairy milk and eggs (milk, milk powder, eggs, and a total of seven items), legumes (bean sprouts, tofu, soy milk), fruits (pit fruits, stone fruits, nuts, and a total of six categories), vegetables (dark-colored vegetables, light-colored vegetables, and mushrooms and edible fungi), and beverages (carbonated beverages, fruit juices, water, and honey water) in the past three months. The intake amount is defined as the total actual consumption per eating occasion, encompassing 40 items. Each item includes six options: “less than 50g, 50-100g, 100-150g, 150-200g, 200-250g, and over 250g”, corresponding to scores of 1 to 6. The total score ranges from 40 to 240 points, with higher scores indicating greater intake amounts. The intake frequency also corresponds to 40 items, each with ten options: “never, less than once a month, 1–3 times a month, 1–2 times a week, 3–4 times a week, 5–6 times a week, once a day, twice a day, three times a day, or more than three times a day”, corresponding to scores of 0 to 9. The total score ranges from 0 to 360 points, with higher scores indicating greater intake frequency. The Cronbach’s alpha coefficient for this survey in the present study is 0.926.

The self-designed exercise questionnaire, based on a literature review and the purpose of this study (20, 21), includes 13 items, including the average daily sitting time and average weekly exercise time in the past 3 months, with a Cronbach’s alpha coefficient of 0.855.

The sleep questionnaire is based on the PSQI self-assessment (for the past month) (22), which comprises 19 items across 7 dimensions. A higher score signifies poorer sleep quality. A PSQI score greater than 7 suggests a sleep quality issue. The Cronbach’s alpha coefficient is 0.849.

The medication questionnaire documents the age of use for antibiotics, probiotics, and vitamin D, with a Cronbach’s alpha coefficient of 0.808.

The psychological questionnaire employs the SCARED scale (for the past month) (23), which consists of 41 items and 5 major anxiety factors. The total score of ≥23 indicates the presence of anxiety, and the reliability of the measurement is supported by a Cronbach’s alpha coefficient of 0.862.

### 2.3 Specimen collection, DNA extraction, and sequencing

Fecal specimens obtained from ISS-derived subjects were preserved in Boyou TM 11901–50 fecal nucleic acid preservation solution (Shanghai Boyu Biotechnology Co., Ltd, https://www.shbio.com/products/3033) at ambient temperature for a maximum of 14 days or at -20°C and -80°C for extended storage. Although no specific experiments were conducted to validate the potential impact of this preservation solution on 16S rRNA sequencing, DNA quality was assessed prior to PCR amplification. The results showed that DNA extracted from the preserved samples met the quality requirements for downstream PCR and sequencing, with no significant degradation or inhibition observed. This preservation solution has been widely used in similar studies without reported negative impacts on sequencing results (24). Samples were transported to the laboratory within 2–3 days and stored at -80°C. DNA extraction was performed utilizing the CTAB method. Extracted DNA quality was assessed via agarose gel electrophoresis, and the diluted DNA served as a template for PCR amplification of the V3-V4 region of the 16S rRNA gene. The primers used were 341F (5’-CCTAYGGGRBGCASCAG-3’) and 806R (5’-GGACTACNNGGGTATCTAAT-3’) (25, 26). PCR amplicons were purified using agarose gel electrophoresis and a Qiagen kit, followed by sequencing using the NEBNext^®^ Ultra™ II DNA Library Prep Kit on the NovaSeq6000 platform. Sequencing data were processed using FLASH for concatenation, fastP for quality control, and Vsearch for chimera removal to generate effective tags (27). The DADA2 module of QIIME2 software was utilized for denoising and ASV screening, with the Silva138.1 database employed for species annotation (28).

### 2.4 Bioinformatics analysis

Bioinformatics analysis was conducted using the ASV table from QIIME2. Alpha diversity was assessed using the Kruskal-Wallis test and rarefaction curves (cutoff=32345) to evaluate ASV abundance, diversity, and evenness. Species richness was quantified using Observed_species and Chao 1 indices, where higher values indicated greater species richness (29). Diversity was assessed using the Shannon and Simpson indices, with higher values indicating greater diversity (30). Evenness was evaluated using Pielou’s index and dominance index, where lower values indicated better community species evenness (31). Species accumulation box plots were used to assess species richness and the emergence rate of novel ASVs, while Relative Abundance bar charts displayed the composition and proportion of gut microbiota. Differential microbiota between the ISS-2SD and ISS-3SD groups were identified using the LEfSe method, incorporating Kruskal-Wallis, Wilcoxon tests, and LDA analysis (LDA score threshold=2.5). A genus-level species evolutionary tree was constructed, and representative sequences of the top 100 genera were obtained through multiple sequence alignment. Spearman correlation analysis was performed to examine the relationship between gut microbiota and environmental factors. The corr.test function from the Psych package in R software was used to calculate Spearman correlation coefficients and test for significance. The Pheatmap function from the Pheatmap package was used for visualization analysis, generating a correlation heatmap. Statistical significance was defined as *P*<0.05. The microbiome sequencing data generated in this study are available in the CNGB Sequence Archive (CNSA) of China National GeneBank DataBase (CNGBdb) under accession number CNP0005704 [https://db.cngb.org/search/?q=CNP0005704].

### 2.5 Data statistics and analysis

General information and data related to gut microbiota were entered and analyzed using IBM SPSS Statistics 27.0 statistical software. General information is described using frequency, percentage, mean, standard deviation, median, and quartiles; Quantitative data were subjected to normality tests using Kolmogorov Smirnov (K-S). Independent sample t-test analysis was used for data that followed a normal distribution, while Mann Whitney U test was used for data that did not follow a normal distribution; Qualitative data is analyzed using chi square test. The test level α=0.05, *P*<0.05 indicates that the difference is statistically significant.

### 2.6 Ethical considerations and quality control

The study was approved by the Medical Ethics Committee of West China Second University Hospital, Sichuan University (Ethical Code: Medical Research 2022 Lun Approval No. 110), with all participants giving informed consent. The research followed principles of informed consent, confidentiality, safety, and benefit. Quality control was strictly maintained throughout the research process.

## 3 Results

### 3.1 General

In this study, the male to female ratio of ISS children was moderate (29/29), all of whom were school-age children with an average age of (9.38 ± 1.80) years and an average height of (120.99 ± 9.60) cm. Children were divided into ISS-2SD group and ISS-3SD group based on their short stature at the time of fecal collection. There were statistically significant differences in age (*P*=0.006), height (*P*=0.002), weight (*P*=0.014), family type (*P*=0.003), meat frequency (*P*=0.008), milk and egg intake (*P*=0.026), fruit frequency (*P*=0.040), and beverage intake (*P*=0.023) between the two groups, while there were no statistically significant differences in other indicators (*P*>0.05). The general information survey results are shown in **Table 1**, and the factors related to gut microbiota are shown in **Table 2**.

**Table 1:** General information survey results.

Taxonomy		ISS(*n*=58)	ISS -2SD(*n*=34)	ISS -3SD(*n*=24)	*χ2/t/Z*	*P*
		*n(*%)/(*x* ± *s)*				
ISS Children’s Information						
Gender					3.317^a^	0.069
	Male	29 (50)	13 (38.2)	15 (44.1)		
	Female	29 (50)	21 (61.7)	9 (26.4)		
Age		9.38 ± 1.80	9.92 ± 1.50	8.61 ± 1.94	2.880^b^	0.006
Height(cm)		120.99 ± 9.60	124.36 ± 7.25	116.23 ± 10.62	3.255^b^	0.002
Height SDS		-2.79 ± 0.66	-2.27 ± 0.15	-3.53 ± 0.29	19.435^b^	<0.001
Weight(kg)		23.14 ± 5.74	24.67 ± 4.87	20.97 ± 6.28	2.526^b^	0.014
BMI		15.56 ± 2.01	15.85 ± 1.87	15.13 ± 2.16	1.340^b^	0.186
Genetic target height(cm)		163.86 ± 7.35	157.68 ± 27.39	164.38 ± 5.95	-1.175^b^	0.245
Birth length(cm)		49.84 ± 2.28	49.74 ± 2.38	50.00 ± 2.19	-0.431^b^	0.668
Birth weight(kg)		3.29 ± 0.24	3.15 ± 0.38	3.10 ± 0.41	0.524^b^	0.602
Delivery method					-0.439^c^	0.661
	Natural birth	31 (53.4)	19 (55.8)	12 (50.0)		
	Caesarean section	27 (46.6)	15 (44.2)	12 (50.0)		
Parity (times)					-0.736^c^	0.462
	1	41 (51.3)	25 (73.5)	16 (66.6)		
	2	15 (18.8)	9 (26.4)	6 (25.0)		
	3	2 (2.5)	0 (0.0)	2 (8.4)		
Stool shape in the past 3 months					-0.099^c^	0.921
	Type 1: Nut shaped	5 (6.3)	2 (5.9)	3 (12.5)		
	Type 2: Dry hard	15 (18.8)	8 (23.5)	7 (29.2)		
	Type 3: wrinkled	12 (15.0)	10 (29.4)	2 (8.3)		
	Type 4: Banana shaped	22 (27.5)	12 (35.3)	10 (41.7)		
	Type 5: Soft and Soft	3 (3.8)	2 (5.9)	1 (4.2)		
	Type 6: Slightly shaped	1 (1.3)	0 (0.0)	1 (4.2)		
Feeding history	Breastfeeding Duration (Months)	7.72 ± 4.45	7.09 ± 4.53	8.63 ± 4.27	-1.304^b^	0.198
	Complementary food month	6.42 ± 1.43	6.47 ± 1.56	6.35 ± 1.23	0.302^b^	0.763
Family information						
Family type					8.929^a^	0.003
	Nuclear family	35 (60.3)	26 (76.5)	9 (37.5)		
	Joint family	23 (39.7)	8 (23.5)	15 (62.5)		
Per capita monthly Income (¥)					6.812^a^	0.078
	0-6000	18 (31.0)	9 (26.5)	9 (37.5)		
	6001-1000	18 (31.0)	9 (26.5)	9 (37.5)		
	10001-2000	12 (20.7)	11 (32.4)	1 (4.2)		
	>20000	10 (17.2)	5 (14.7)	5 (20.8)		
Father’s age (years)		39.31 ± 5.06	39.24 ± 5.08	39.42 ± 5.16	-0.133^b^	0.895
Mother’s age (years)		36.72 ± 4.46	37.09 ± 4.45	36.21 ± 4.53	0.736^b^	0.465
Father’s height (cm)		171.56 ± 4.08	162.37 ± 28.98	159.80 ± 33.40	0.310^b^	0.758
Mother’s height (cm)		156.60 ± 4.61	151.81 ± 26.99	150.65 ± 32.04	0.149^b^	0.882
Father’s level of education					3.093^a^	0.378
	Primary school or below	2 (3.4)	1 (2.9)	1 (4.2)		
	Middle school	8 (13.8)	3 (8.8)	5 (20.8)		
	High school	19 (32.8)	10 (29.4)	9 (37.5)		
	Bachelor’s degree or above	29 (50.0)	20 (58.8)	9 (37.5)		
Mother’s level of education					1.965^a^	0.580
	Primary school or below	1 (1.7)	1 (2.9)	0 (0.0)		
	Middle school	8 (13.8)	5 (14.7)	3 (12.5)		
	High school	26 (44.8)	13 (38.2)	13 (54.2)		
	Bachelor’s degree or above	23 (39.7)	15 (44.1)	8 (33.3)		
Mother’s pregnancy situation						
Pregnancy age (years)		27.71 ± 4.30	27.56 ± 4.28	27.92 ± 4.42	-0.309^b^	0.758
Pre pregnancy diseases					2.935^a^	0.087
	No	56 (96.6)	34 (100.00)	22 (91.6)		
	Yes	2 (3.4)	0 (0.00)	2 (8.3)		
Pregnancy related illnesses					0.816^a^	0.366
	No	51 (87.9)	31 (91.1)	20 (83.3)		
	Yes	7 (12.1)	3( 8.8)	4 (16.6)		
Pregnancy medication					0.834^a^	0.361
	No	55 (94.8)	33 (97.0)	22 (91.6)		
	Yes	3 (5.2)	1 (2.9)	2 (8.3)		
Radiation during pregnancy					0.718^a^	0.397
	No	57 (98.3)	33 (97.0)	24 (100.0)		
	Yes	1 (1.7)	1 (2.9)	0 (0.0)		

^a^Chi square test χ^2^-value, ^b^independent sample t-test t-value, ^c^Mann Whitney U test Z-value.

**Table 2:** Investigation results of factors related to intestinal microbiota in ISS children.

Taxonomy		ISS (n=58)	ISS -2SD (*n*=34)	ISS -3SD (*n*=24)	*t/z*	*P*
		(*x* ± *s)*				
Diet (Meal Intake and Frequency Past 3 Months*)						
Total staple frequency		25.79 ± 4.34	25.85 ± 4.89	25.71 ± 3.51	0.124	0.902
Total staple intake		13.79 ± 3.66	13.88 ± 3.74	13.67 ± 3.61	0.219	0.827
Meat frequency		19.52 ± 5.23	21.03 ± 5.20	17.38 ± 4.58	2.769	0.008
Meat intake		14.24 ± 4.80	14.74 ± 5.49	13.54 ± 3.61	0.931	0.356
Milk and egg frequency		16.57 ± 3.33	17.00 ± 2.89	15.96 ± 3.85	1.177	0.244
Milk and egg intake		14.57 ± 3.83	15.50 ± 3.75	13.25 ± 3.61	2.283	0.026
Legumes frequency		4.76 ± 2.33	5.15 ± 2.36	4.21 ± 2.23	1.526	0.133
Legumes intake		4.55 ± 1.93	4.47 ± 2.22	4.67 ± 1.46	-0.378	0.707
Fruit frequency		15.03 ± 5.85	16.35 ± 5.69	13.17 ± 5.66	2.105	0.040
Fruit intake		11.16 ± 5.39	11.29 ± 5.70	10.96 ± 5.03	0.232	0.818
Vegetable frequency		10.79 ± 4.34	11.09 ± 4.11	10.38 ± 4.71	0.612	0.543
Vegetable intake		4.74 ± 2.00	4.86 ± 2.28	4.58 ± 1.56	0.501	0.618
Beverage frequency		11.07 ± 2.94	11.59 ± 3.06	10.33 ± 2.65	1.625	0.110
Beverage intake		8.88 ± 4.13	9.82 ± 4.73	7.54 ± 2.62	2.348	0.023
Exercise (3-Month Avg. Daily Sitting & Weekly Exercise Time)						
Outdoor exercise time (h)		1.58 ± 0.76	1.59 ± 0.81	1.58 ± 0.70	0.010	0.992
Indoor activity time (h)		1.21 ± 0.49	1.22 ± 0.47	1.19 ± 0.52	0.244	0.808
Sitting time (h)		6.12 ± 3.82	5.77 ± 3.94	6.61 ± 3.67	-0.821	0.415
Exercise time (h)		3.88 ± 2.40	4.06 ± 2.48	3.62 ± 2.32	0.676	0.502
Aerobic exercise time (h)		2.40 ± 1.55	2.48 ± 1.55	2.30 ± 1.58	0.441	0.661
Anaerobic exercise time (h)		0.87 ± 0.89	0.94 ± 0.92	0.76 ± 0.85	0.783	0.437
Resistance exercise time (h)		0.61 ± 0.78	0.63 ± 0.78	0.57 ± 0.81	0.312	0.756
Medication (earliest sub drug use age group)						
Antibiotic use age					-1.374**	0.169
	Never	9 (15.5)	2 (5.9)	7 (29.2)		
	<6 months old	6 (10.3)	5 (14.7)	1 (4.2)		
	From 7 months to 1 year old	28 (48.3)	17 (50.0)	11 (45.8)		
	>1.1 years old	15 (25.9)	10 (29.4)	5 (20.8)		
Probiotics use age					-0.462**	0.644
	Never	54 (93.1)	30 (88.3)	24 (100.0)		
	<6 months old	1 (1.7)	1 (2.9)	0 (0.0)		
	From 7 months to 1 year old	2 (3.4)	2 (5.9)	0 (0.0)		
	>1.1 years old	1 (1.7)	1 (2.9)	0 (0.0)		
Vitamin D use age					-0.127**	0.899
	Never	2 (3.4)	2 (5.9)	0 (0.0)		
	<6 months old	44 (75.9)	24 (70.6)	20 (83.3)		
	From 7 months to 1 year old	10 (17.2)	7 (20.6)	3 (12.5)		
	>1.1 years old	2 (3.4)	1 (2.9)	1 (4.2)		
Sleep (past month)						
PSQI score		4.97 ± 4.61	5.50 ± 5.10	4.21 ± 3.79	1.052	0.297
Psychology (past month)						
SCARED score		12.24 ± 7.60	13.18 ± 7.91	10.92 ± 7.08	1.118	0.268

*The intake amount is defined as the weight (in grams) of food consumed per eating occasion., the intake frequency includes “never, less than once a month, 1–3 times a month, 1–2 times a week, 3–4 times a week, 5–6 times a week, once a day, twice a day, three times a day, or more than three times a day”. **Mann-Whitney *U test Z-value.*

### 3.2 Sequencing data results and quality assessment

**Figure 1:**
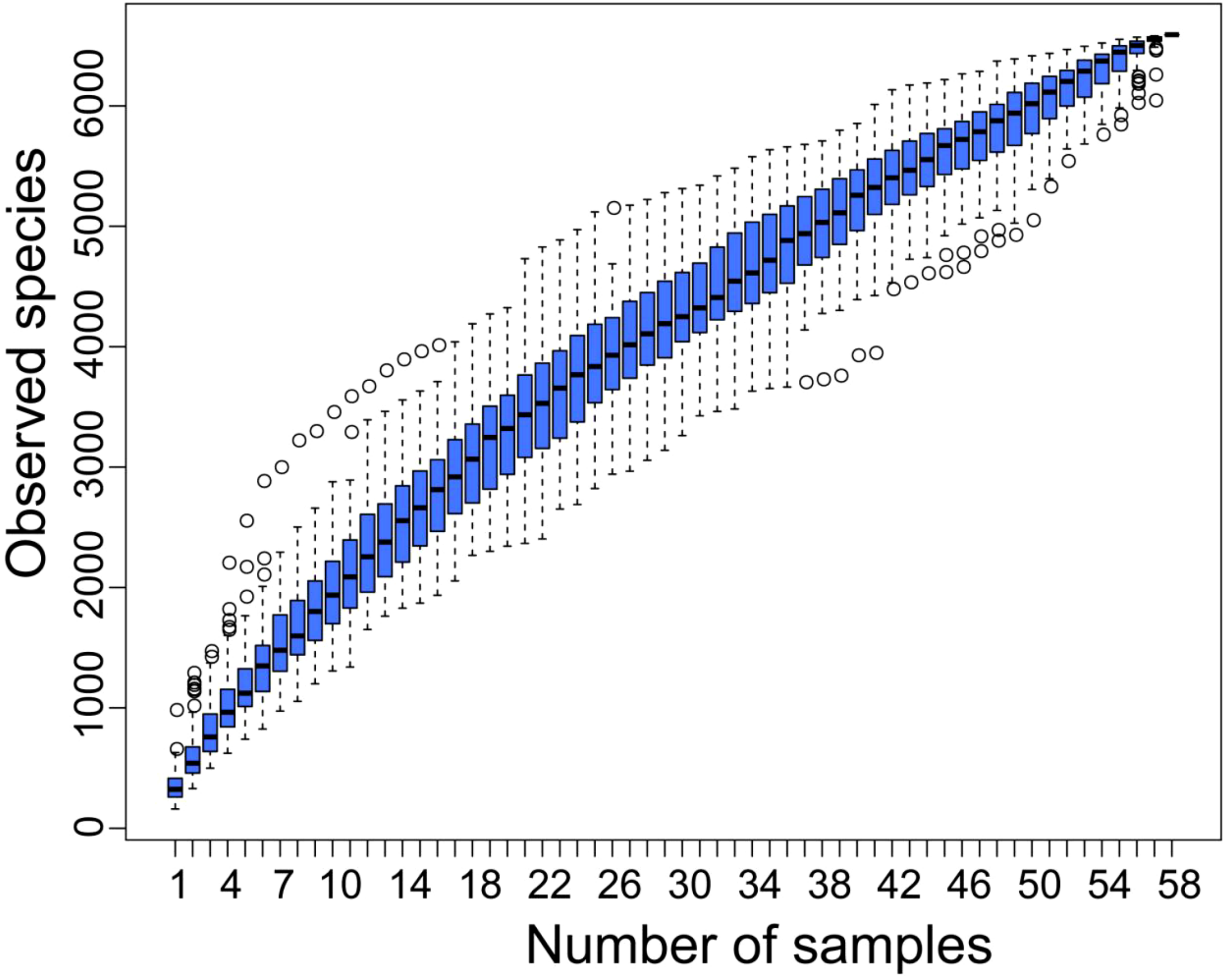
Cumulative box chart of alpha diversity species. The horizontal axis represents the sample size; The vertical axis represents the number of ASVs after sampling.

To ensure the accuracy of the analysis, the raw sequencing data was concatenated, filtered, and processed with DADA2 denoising to exclude sequences with readings below 5, resulting in ASV (32). This study analyzed a total of 58 samples and 6592 ASVs were obtained through ASV clustering, with a proportion of 99.2% annotated to the boundary level, covering 44 phyla (98.3%), 111 classes (98.1%), 256 orders (96.8%), 435 families (92.9%), 1060 genera (76.0%), and 849 species (15.8%). The analysis of the cumulative box plot of species in this study showed that the position of the box plot first sharply increased and then gradually flattened, indicating that the sample size of this study was sufficient (**Figure 1**).

### 3.3 Alpha diversity analysis

**Figure 2:**
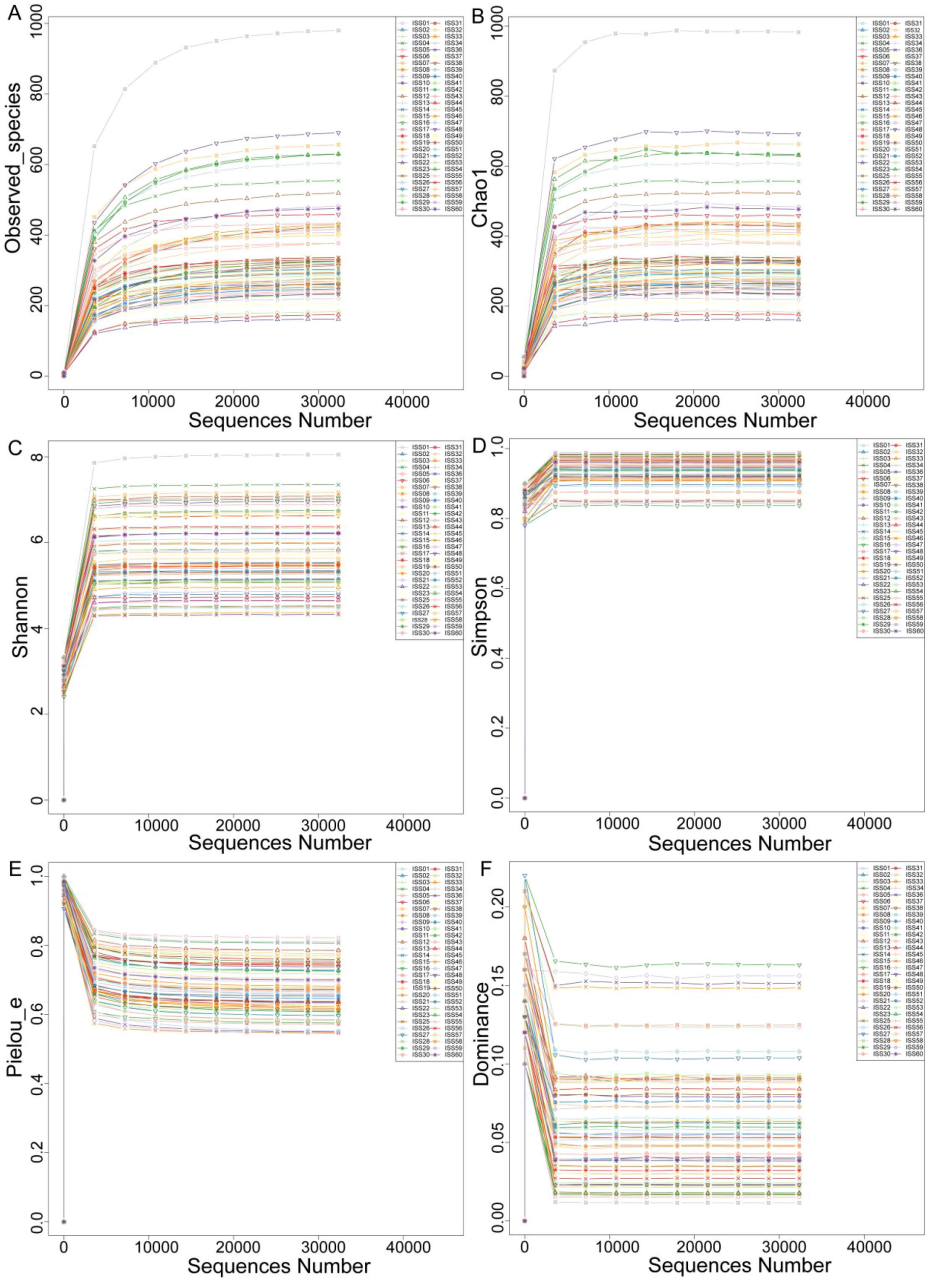
Rarefaction curve. The horizontal axis represents the sequencing data volume, and the vertical axis represents the corresponding alpha diversity index. **(A)** Observed_species, **(B)** Chao 1, **(C)** Shannon index, **(D)** Simpson index, **(E)** Pielou’s index, **(F)** Dominance index.

**Figure 3:**
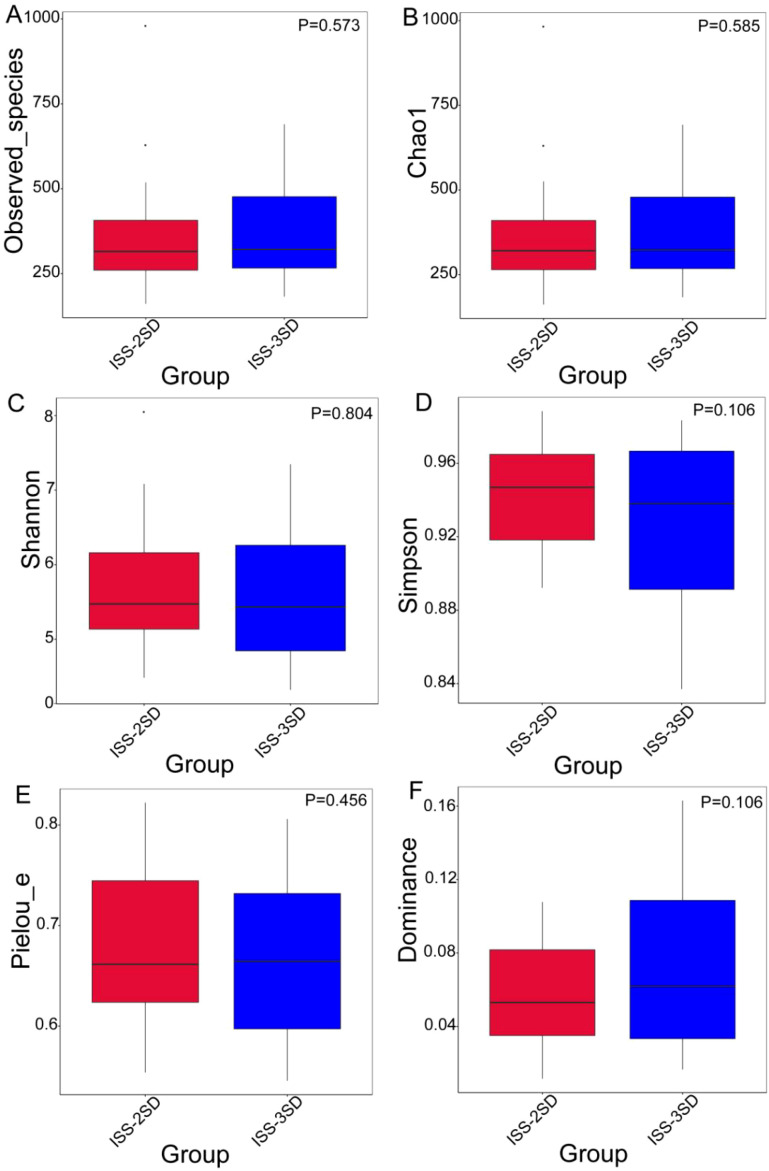
Cumulative box chart of alpha diversity species. The chart shows the diversity of bacterial species in two subgroups: ISS-2SD (n=34) and ISS-3SD (n=24). The horizontal axis represents grouping, and the vertical axis represents the corresponding alpha index values. **(A)** Observed_species, **(B)** Chao 1, **(C)** Shannon index, **(D)** Simpson index, **(E)** Pielou’s index, **(F)** Dominance index.

The rarefaction curves for alpha diversity exhibit a plateau, signifying that the sequencing data adequately encompasses the predominant microbial diversity within the samples. This observation implies that further sequencing would yield minimal gains in diversity indices, including the Shannon and Simpson indices. Consequently, the current sequencing depth is considered sufficient for a thorough characterization of the gut microbiota in this investigation (**Figure 2**). The analysis of differences in alpha diversity index between ISS children with different levels of dwarfism showed that there were no statistically significant differences in the Observed_species (*P*=0.573) and Chao 1 (*P*=0.585) representing species richness, Shannon index (*P*=0.804) and Simpson index (*P*=0.106) representing diversity, and Pielou’s index (*P*=0.456 and dominance index (*P*=0.106) representing evenness between the ISS-2SD and ISS-3SD groups (*P*>0.05) (**Figure 3**).

### 3.4 Differences in bacterial composition between groups

**Figure 4:**
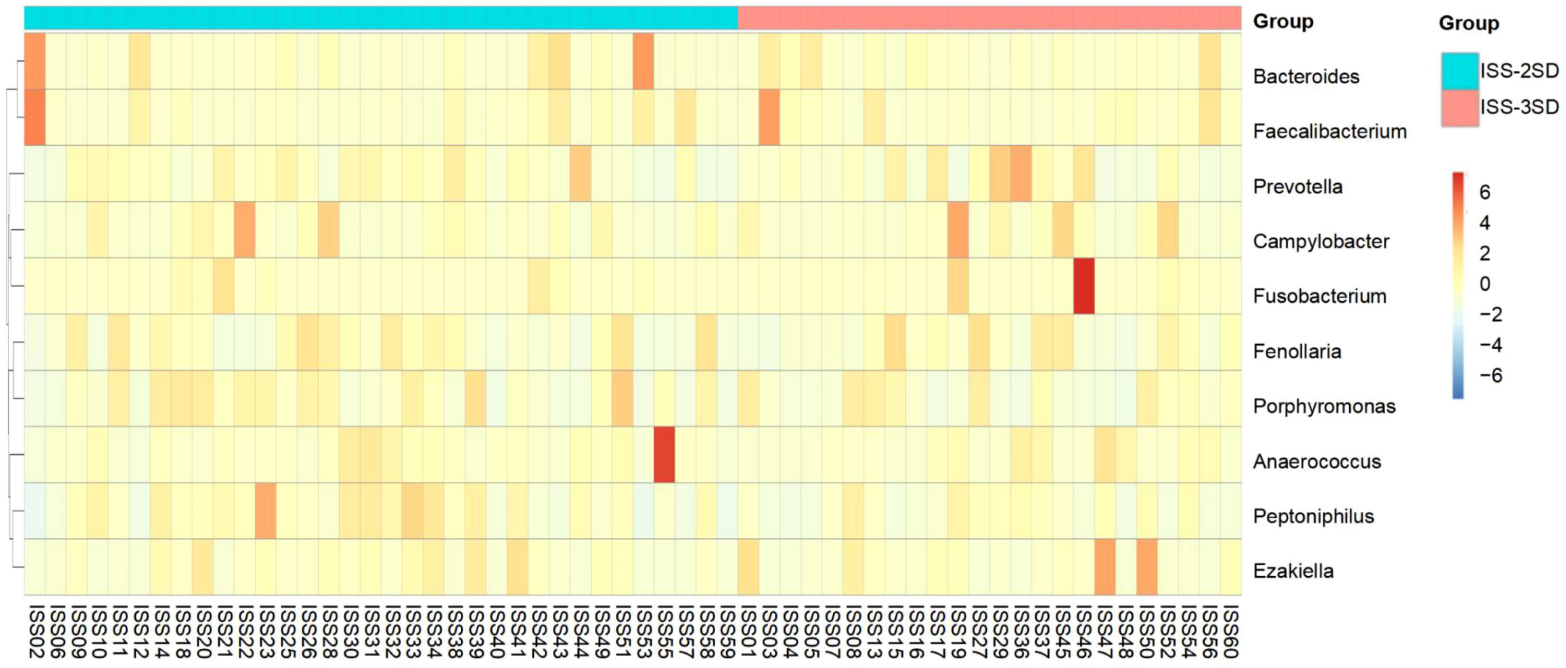
Heatmap analysis of the top 10 gut microbiota genera in ISS-2SD (*n*=34) and ISS-3SD (*n*=24) groups.

The heatmap analysis of gut microbiota at the genus level for children in the ISS-2SD and ISS-3SD groups showed that the top 10 ranked genera were *Bacteroides, Faecalibacterium, Prevotella, Campylobacter, Fusobacterium, Fenollaria, Porphyromonas, Anaerococcus, Peptoniphilus, and Ezakiella* (**Figure 4**).

**Figure 5:**
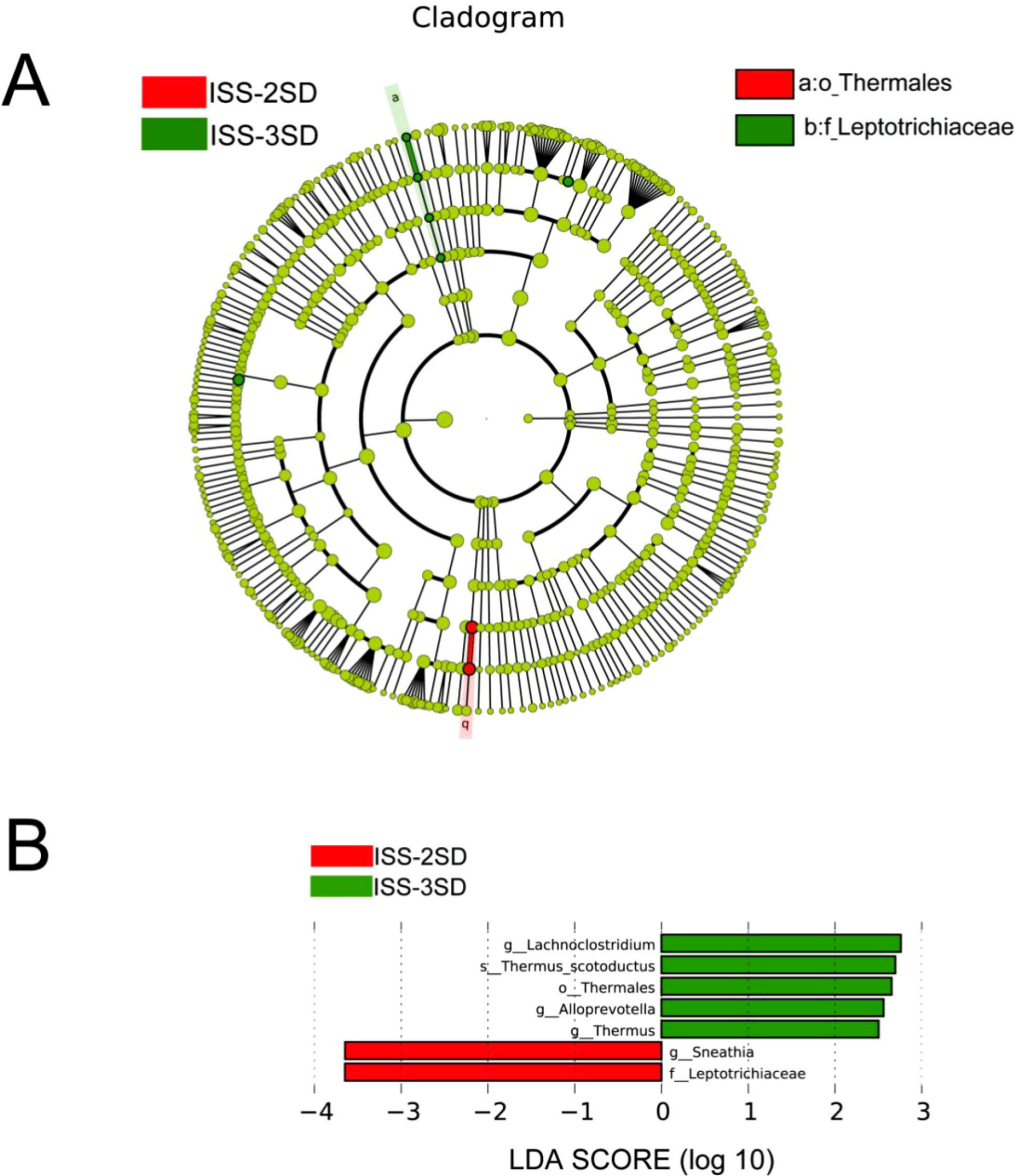
Analysis of inter group microbial diversity. **(A)** Evolutionary diagram with circle sizes indicating abundance, colors for species differentiation, red and green nodes for key microbial groups, and species names in the legend. **(B)** LDA scores for species in ISS-2SD (*n*=34) and ISS-3SD (*n*=24) groups, with colors for group distinction and bar lengths for species contribution, showing only species with LDA ≥2.5.

The LEfSe method was employed to construct the evolutionary branch diagram (**Figure 5A**) and the LDA value distribution histogram (**Figure 5B**). These visualizations highlight significant differences in the dominant microbial communities among ISS children with varying degrees of short stature, evident at the family, genus, and species taxonomic levels. Specifically, the ISS-2SD group exhibited two predominant bacterial groups: *Leptotrichiaceae* (LDA=3.63, *P*=0.033) and *Sneathia* (LDA=3.65, *P*=0.046). In contrast, the ISS-3SD group featured five distinct dominant taxa: *Lachnoclostridium* (LDA=2.76, *P*=0.016), *Thermus scotoductus* (LDA=2.70, *P*=0.036), *Thermales* (LDA=2.50, *P*=0.036), *Alloprevotella* (LDA=2.56, *P*=0.006), and *Thermus* (LDA=2.65, *P*=0.036).

### 3.5 Correlation analysis of gut microbiota

**Figure 6:**
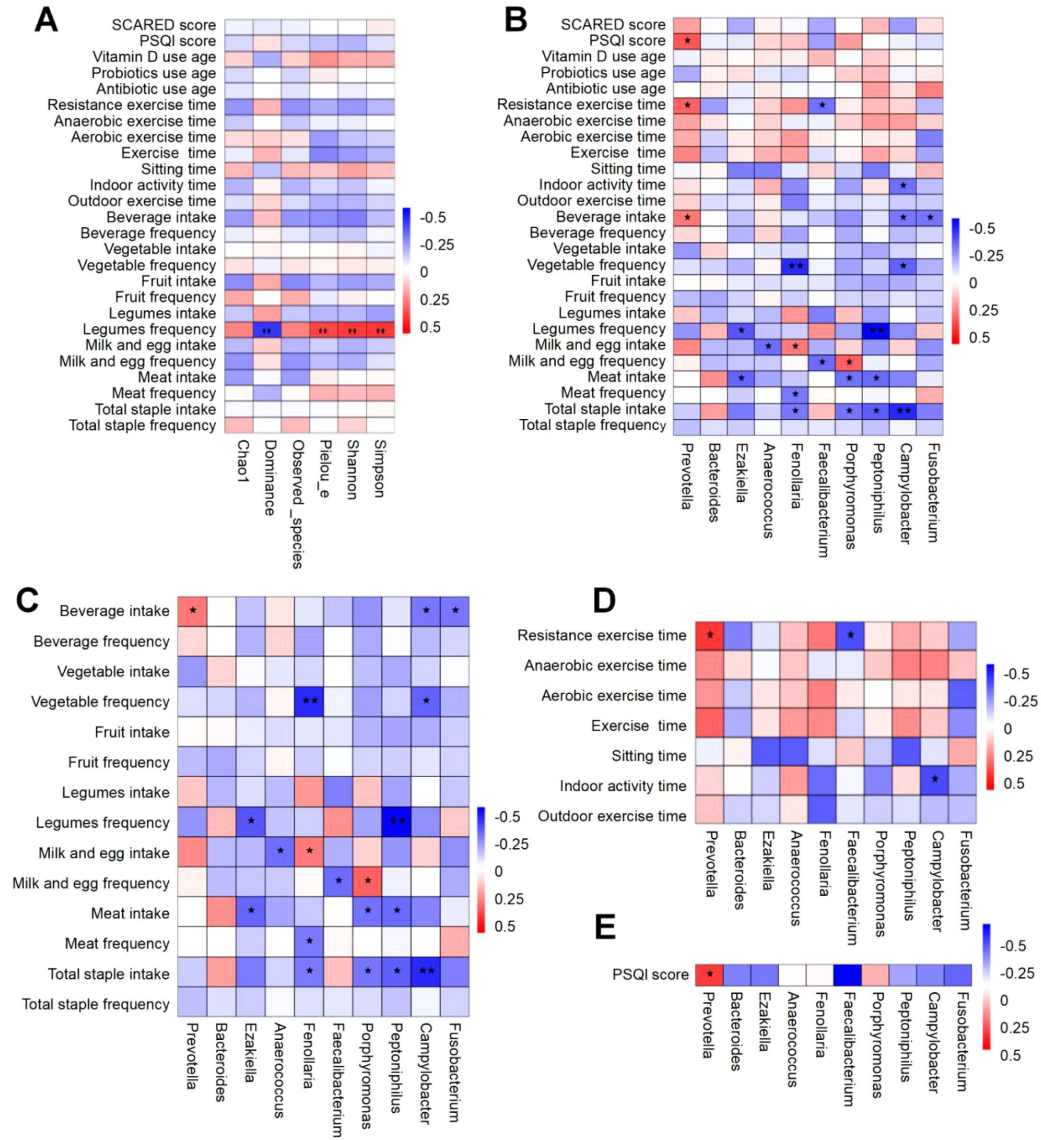
Spearman correlation heatmap of factors associated with gut microbiota in ISS children. The vertical axis represents environmental factors, while the horizontal axis corresponds to microbial species. Heatmap values indicate Spearman’s correlation coefficients (ranging from -1 to 1), where negative correlations (r < 0) and positive correlations (r > 0) are depicted. Significance levels are denoted as follows: “*” for *P*<0.05 and “**” for *P*<0.01. **(A)** Correlation between gut microbiota diversity and lifestyle factors. **(B)** Correlation analysis of the top 10 genus-level microbes with overall lifestyle factors. **(C)** Correlation analysis of the top 10 genus-level microbes with dietary factors. **(D)** Correlation analysis of the top 10 genus-level microbes with exercise-related factors. **(E)** Correlation analysis of the top 10 genus-level microbes with sleep-related factors.

To elucidate the determinants influencing the gut microbiota of children afflicted with ISS, we employed spearman’s rank correlation analysis to study the correlation between the alpha diversity of the gut microbiota and the lifestyle factors of ISS children. Additionally, we analyzed the correlation between the top 10 gut microbial genera at the genus level and the lifestyle factors of ISS children, obtaining the correlation coefficients and the significant *P*-values between these factors. The analysis results of the correlation between gut microbiota alpha diversity and the lifestyle of children with ISS show that gut microbiota alpha diversity is significantly correlated with diet (**Figure 6A**). Specifically, the Shannon and Simpson indices representing species diversity are positively correlated with legume frequency(r=0.372, *P*=0.004, and r=0.379, *P*=0.003); the Pielou’s index representing uniformity is positively correlated with legumes frequency(r=0.354, *P*=0.006), the Dominance index is negatively correlated with legume frequency(r=-0.379, *P*=0.003).

Correlation analysis between the top 10 gut microbial genera at the genus level and the lifestyle of children with ISS demonstrated significant associations between diet, exercise, and sleep patterns with the gut microbiota(**Figure 6B**). Specifically, the correlation between diet and gut microbiota is shown in **Figure 6C**: *Prevotella* was significantly positively correlated with beverage intake (r=0.263, *P*=0.046). *Ezakiella* was significantly negatively correlated with meat intake and legume frequency (r=-0.297, *P*=0.024 and r=-0.324, *P*=0.013). *Anaerococcus* was significantly negatively correlated with milk and egg intake (r=-0.278, *P*=0.034). *Fenollaria* was significantly negatively correlated with total staple intake, meat frequency, and vegetable frequency (r=-0.259, *P*=0.050; r=-0.259, *P*=0.050 and r=-0.409, *P*=0.001). *Faecalibacterium* was significantly negatively correlated with milk and egg frequency (r=-0.282, *P*=0.032). *Porphyromonas* was significantly negatively correlated with total staple intake and meat intake (r=-0.268, *P*=0.042 and r=-0.271, *P*=0.039), while it was significantly positively correlated with milk and egg frequency (r=0.304, *P*=0.020). *Peptoniphilus* was significantly negatively correlated with total staple intake, meat intake, and legume frequency (r=-0.312, *P*=0.017; r=-0.287, *P*=0.029 and r=-0.479, *P*<0.001). *Campylobacter* was significantly negatively correlated with total staple intake, vegetable frequency, and beverage intake (r=-0.410, *P*=0.001; r=-0.302, *P*=0.021 and r=-0.260, *P*=0.048). *Fusobacterium* was significantly negatively correlated with beverage intake (r=-0.273, *P*=0.038).

Furthermore, the correlation analysis results between gut microbiota and exercise are shown in **Figure 6D**: *Prevotella* was significantly positively correlated with resistance exercise time (r=0.304, *P*=0.020). *Faecalibacterium* was significantly negatively correlated with resistance exercise time (r=-0.275, *P*=0.037). *Campylobacter* was significantly negatively correlated with indoor activity time (r=-0.278, *P*=0.035).

Additionally, the correlation analysis between sleep and gut microbiota indicates (as shown in **Figure 6E**): *Prevotella* is significantly positively correlated with the PSQI score (a measure of sleep quality) (r=0.323, *P*=0.013).

## 4 Discussion

### 4.1 Gut microbiota varies among ISS with different levels of dwarfism

This study showed that there was no significant difference in the alpha diversity of the intestinal microbiota among children with different degrees of short stature, but there were differences in the dominant microbiota, indicating that although the overall diversity index was similar, the specific composition of the microbiota was different among children with different degrees of short stature. Compared with previous studies, this study provides more detailed information on the composition of the microbiota and distinguishes the dominant microbiota in children with ISS-2SD and ISS-3SD (8; Lin 9). Revealed notable disparities in the prevailing microbial communities between the ISS-2SD and ISS-3SD cohorts. Specifically, the ISS-2SD group exhibited a dominance of *Leptotrichiaceae* and *Sneathia*, whereas the ISS-3SD group was characterized by a prevalence of *Lachnoclostridium*, *Thermus scotoductus*, *Thermales*, *Alloprevotella*, and *Thermus* as the predominant taxa. These differences may signify potential functional variations within the gut microbiota of pediatric subjects exhibiting varying degrees of short stature.

Initially, *Leptotrichiaceae* and *Sneathia* demonstrated significant enrichment within the ISS-2SD group. Prior investigations have established a correlation between *Sneathia* and gut health, as well as maternal microbial transmission, particularly in infants delivered via cesarean section, where *Sneathia* may influence early gut microbiota colonization through the maternal vaginal microbiota (33). Furthermore, certain members of the *Leptotrichiaceae* family have been implicated in oral and gut health, potentially impacting host metabolism and development by modulating local immune responses (34).

In contrast, *Lachnoclostridium*, which was significantly enriched in the ISS-3SD group, is a key producer of short-chain fatty acids (SCFAs). It can ferment polysaccharides to yield metabolites such as butyrate and acetate, which positively influence the proliferation of intestinal epithelial cells and enhance gut barrier function (34). Furthermore, *Thermus scotoductus* and *Thermus* are thermophilic bacteria, and while their specific roles in the gut remain unclear, studies suggest that thermophiles may regulate host metabolism and energy balance under extreme conditions (35). *Alloprevotella*, in contrast, is strongly associated with high-fiber diets and may influence growth and development by modulating host immune responses and metabolic health (36).

These findings suggest that the degree of short stature in ISS children may be closely associated with functional differences in their gut microbiota. Future research should further elucidate the specific metabolic functions of these dominant microbial taxa and their potential impact on host growth and development. In particular, interventions targeting dietary modifications or probiotics administration to modulate the gut microbiota may offer novel therapeutic strategies for the management of ISS.

### 4.2 Dietary factors correlate with gut microbiota alpha diversity in ISS

This study assessed the association between gut microbiota alpha diversity and the lifestyle of children with ISS and found that gut microbiota alpha diversity is significantly correlated with diet, particularly the frequency of legume intake. Specifically, the Shannon index and Simpson index, which represent species diversity, were significantly positively correlated with the frequency of legume intake, and the Pielou index, which represents evenness, was also positively correlated with the frequency of legume intake. In contrast, the Dominance index, which represents dominance, was negatively correlated with the frequency of legume intake. These results suggest that legume intake may positively impact the gut health of children with ISS by increasing the diversity and evenness of the gut microbiota, at the same time, the frequency of legumes may promote the balanced development of microbial communities by reducing the dominance of certain species.

Legumes characterized by their high content of dietary fiber and various phytochemicals, which have been demonstrated to promote the diversity and functionality of the gut microbiota, thereby contributing to improved health outcomes (37, 38). Prior research has established a positive correlation between dietary fiber intake and the diversity and richness of the gut microbiota, particularly in increasing the abundance of beneficial bacteria such as *Bifidobacteria* and *Lactobacilli* (39). This is consistent with our research findings that the frequency of legume intake is positively correlated with Shannon and Simpson indices, indicating that legumes may promote the diversity of gut microbiota by providing abundant dietary fiber. Secondly, phytochemicals in legumes, such as polyphenolic compounds, have also been shown to regulate the gut microbiota. A study found that polyphenolic compounds can improve the uniformity of gut microbiota by inhibiting the growth of harmful bacteria and promoting the proliferation of beneficial bacteria (40). This is consistent with the positive correlation between Pielou index and legume intake frequency in our research results, indicating that polyphenolic compounds in legumes may play an important role in regulating the evenness of gut microbiota. Additionally, the negative correlation between legume intake and the Dominance index suggests that legume consumption may attenuate the overgrowth of certain dominant bacterial groups, thereby maintaining the equilibrium of the gut microbiota. This observation is consistent with recent findings indicating that high-fiber diets can reduce the abundance of certain potentially pathogenic bacteria, thus diminishing the dominance within the gut microbiota (41).

Therefore, the results of this study indicate a significant and positive correlation between the frequency of legume intake and the diversity and evenness of the gut microbiota in children with ISS, while a negative correlation was observed with dominance. These findings suggest that increased legume intake may positively impact the gut health of children with ISS by modulating the structure and function of the gut microbiota. Future studies should focus on elucidating the long-term effects of legume intake on the growth and development of children with ISS and exploring the underlying molecular mechanisms.

### 4.3 Intestinal microbiota and associated factors in children with ISS

#### 4.3.1 Correlation of gut microbiota with dietary factors

The correlation between diet and gut microbiota has become an important area of research, as gut microbiota composition is known to play a critical role in host metabolism, immune function, and overall health. In this study, we observed significant correlations between various dietary factors and gut microbiota taxa such as *Prevotella*, *Ezakiella*, *Anaerococcus*, and others, which supports existing research on the diet-microbiota interaction.

To further contextualize these findings, we propose that the observed associations may be mediated by the metabolic activities of these microbial taxa. For instance, *Prevotella* was significantly positively correlated with beverage intake, aligning with studies indicating its association with carbohydrate-rich diets, including sugary or fiber-rich beverages (36, 42). This genus is known for its ability to metabolize complex carbohydrates and fibers, which are abundant in plant-based diets. The positive correlation with beverage consumption may reflect the role of *Prevotella* in fermenting dietary fibers, producing short-chain fatty acids (SCFAs) that benefit host health (43).

On the other hand, *Ezakiella* showed a significant negative correlation with meat intake and legume frequency, suggesting that high-protein diets, particularly those rich in animal products, may suppress its growth (37 The negative correlation with legumes, however, is intriguing and may suggest a unique sensitivity of this genus to specific plant-based proteins or fibers. This could be due to the presence of anti-nutritional factors in legumes, such as lectins or phytates, which may inhibit the growth of certain microbial taxa (44).

Similarly, *Anaerococcus* and *Faecalibacterium* were negatively correlated with milk and egg intake, highlighting the potential adverse effects of high-protein or high-fat diets on beneficial gut bacteria. *Faecalibacterium*, known for its anti-inflammatory properties and role in producing butyrate, is more abundant in individuals consuming plant-based diets (45). The absence of dietary fiber in milk and eggs may further suppress its growth, as high intake of animal products is associated with reduced microbial diversity and increased inflammation (37

*Fenollaria* exhibited significant negative correlations with total staple intake, meat frequency, and vegetable frequency, suggesting that this genus may thrive in environments with limited dietary diversity. Research indicates that low-diversity diets often favor the growth of specific, less-common taxa like *Fenollaria*, while high intake of staple foods and plant-based diets typically promote fiber-degrading bacteria, which may compete with *Fenollaria* (37, 46)

*Porphyromonas* and *Peptoniphilus* showed complex relationships with diet. *Porphyromonas* was negatively correlated with total staple intake and meat intake but positively correlated with milk and egg frequency, reflecting its adaptability to different dietary components (47). *Peptoniphilus* was significantly negatively correlated with total staple intake, meat intake, and legume frequency, indicating a preference for animal-derived proteins (48). Finally, *Campylobacter* and *Fusobacterium* were negatively correlated with beverage intake, suggesting that these genera may be less prevalent in individuals consuming sugary or caffeinated beverages, consistent with studies linking them to low sugar intake (49).

In conclusion, these findings underscore the intricate relationship between diet and gut microbiota, highlighting the need for further research to elucidate the mechanisms underlying these associations. Future studies could explore the role of specific dietary components, such as fiber types or protein sources, in shaping the gut microbiome and their potential implications for host health.

#### 4.3.2 Correlation of gut microbiota with exercise factors

The correlation analysis between gut microbiota and exercise in this study revealed a significant association, highlighting the impact of physical activity on gut microbiota composition. *Prevotella* was significantly positively correlated with resistance exercise time, suggesting that this genus may benefit from anaerobic or high-intensity exercise. This finding aligns with studies indicating that *Prevotella* is associated with diets rich in complex carbohydrates and fibers, which are often linked to active lifestyles (42). Additionally, research by Estaki et al. (50) demonstrated that exercise can increase the abundance of *Prevotella*, potentially due to its role in metabolizing dietary fibers and supporting energy metabolism during physical activity.

To further contextualize these findings, we propose that the positive correlation between *Prevotella* and resistance exercise may be mediated by exercise-induced changes in gut motility and blood flow, which can enhance nutrient availability for microbial fermentation (51). This could explain why *Prevotella*, a genus known for its ability to metabolize complex carbohydrates, thrives in individuals engaging in high-intensity exercise.

In contrast, *Faecalibacterium* was significantly negatively correlated with resistance exercise time. This is intriguing, as *Faecalibacterium prausnitzii* is known for its anti-inflammatory properties and butyrate production, which are generally beneficial for gut health (52). The negative correlation may reflect the impact of high-intensity exercise on microbial diversity, as studies have shown that intense physical activity can temporarily reduce the abundance of certain beneficial bacteria, including *Faecalibacterium* (53). This could be due to exercise-induced stress or changes in gut permeability, which may alter the microbial environment (54).

*Campylobacter* was significantly negatively correlated with indoor activity time, suggesting that this genus may be less prevalent in individuals who engage in more sedentary behaviors. This finding is consistent with research indicating that *Campylobacter* is associated with low physical activity levels and poor dietary habits, such as high sugar intake (49, 53). The reduction in *Campylobacter* abundance with increased indoor activity time may reflect the benefits of even moderate physical activity in promoting a healthier gut microbiota composition.

These findings underscore the complex relationship between exercise and gut microbiota, highlighting the need for further research to elucidate the mechanisms underlying these associations. For example, future studies could explore how different types and intensities of exercise influence specific microbial taxa and their functional roles in gut health. Additionally, the role of exercise-induced changes in gut motility, blood flow, and permeability in shaping the gut microbiome warrants further investigation.

#### 4.3.3 Correlation between gut microbiota and sleep factors

The correlation analysis between sleep and gut microbiota reveals a significant positive correlation between *Prevotella* and the PSQI score, indicating that higher *Prevotella* abundance is associated with poorer sleep quality. Research by Smith et al. (55) demonstrated that gut microbiota diversity and composition are closely linked to sleep quality, with certain microbial taxa, including *Prevotella*, being more abundant in individuals with sleep disorders. This may be due to the production of metabolites such as SCFAs or neurotransmitters that can influence sleep regulation (55). Additionally, studies have shown that *Prevotella* can modulate systemic inflammation, which is known to affect sleep quality. For example, chronic low-grade inflammation associated with higher *Prevotella* abundance may disrupt sleep by altering circadian rhythms or increasing stress responses (56).

To further contextualize these findings, we propose that the relationship between *Prevotella* and sleep quality may be mediated by the gut-brain axis, which plays a critical role in regulating sleep. *Prevotella* has been shown to influence the production of serotonin, a neurotransmitter involved in sleep regulation (57). Changes in serotonin levels due to *Prevotella* abundance could contribute to sleep disturbances, as serotonin is a precursor to melatonin, the hormone responsible for regulating sleep-wake cycles (58). Furthermore, *Prevotella* may affect sleep through its role in modulating the hypothalamic-pituitary-adrenal (HPA) axis, which regulates stress responses and sleep patterns (59).

Additionally, the production of microbial metabolites, such as SCFAs, by *Prevotella* may influence sleep by affecting the central nervous system. SCFAs, particularly butyrate, have been shown to cross the blood-brain barrier and modulate neurotransmitter activity, potentially impacting sleep quality (60). This mechanistic link provides a plausible explanation for the observed correlation between *Prevotella* and poor sleep quality.

In conclusion, the positive correlation between *Prevotella* and PSQI scores highlights the potential role of gut microbiota in sleep regulation. Further research is needed to explore the mechanisms underlying this relationship, including the role of microbial metabolites, immune modulation, and the gut-brain axis in sleep quality. Future studies could also investigate how interventions targeting the gut microbiota, such as probiotics or dietary modifications, may improve sleep outcomes in individuals with sleep disorders.

#### 4.3.4 Correlation of gut microbiota with medication and psychological conditions factors

This study did not find a significant association between the intestinal microbiota and medication and psychological state of children with ISS, probably because children who had used antibiotics or microbiota in the past three months were excluded. The effects of drugs such as antibiotics on the gut microbiota usually last 1 to 3 months, so children who have recently taken the drug were excluded from the study to reduce interference (61). The use of drugs in children with ISS included early antibiotic use and microecological intake, but these factors were not significantly related to the intestinal microbiota due to the completion of drug metabolism.

In terms of psychological status, since children with severe psychological disorders are excluded, and the questionnaire used may not be able to comprehensively measure other psychological problems, future studies need to further explore the relationship between intestinal microbiota and medication and psychological state in children with ISS. For example, future research could incorporate more comprehensive psychological assessments, such as the Child Behavior Checklist (CBCL) or the Strengths and Difficulties Questionnaire (SDQ), to better capture the psychological state of children with ISS (62). Additionally, longitudinal studies could track the long-term effects of medication use, particularly antibiotics, on gut microbiota composition and its potential impact on psychological health (63).

Thus, the maintenance of health and the modulation of the gut microbiota are critically dependent on an active and salubrious lifestyle, encompassing a balanced dietary regimen, regular physical activity, and adequate sleep. Our findings underscore the significance of cultivating optimal lifestyle behaviors in pediatric populations afflicted with ISS. Consequently, collaborative efforts between societal entities and familial units are imperative to establish a supportive milieu that fosters the adoption of salutary behaviors in children. This entails the provision of nutrient-dense sustenance, the promotion of physical engagement, the assurance of sufficient rest and sleep, and the education of children regarding the intrinsic value of a healthful lifestyle. These interventions are instrumental in establishing a robust foundation for the long-term well-being of children with ISS.

## 5 Conclusions and recommendations

This study was the first study in southwest China to explore the intestinal microbiota status of children with different degrees of short stature and its relationship with lifestyle in children with ISS. The results showed that although there was no significant difference in the alpha diversity index of intestinal microbiota between the two groups of children with ISS-2SD and ISS-3SD, there was a significant difference in the composition of the dominant microbiota. The dominant micromicrobiota in the ISS-2SD group were *Leptotrichiaceae* and *Sneathia*, while the ISS-3SD group included five micromicrobiota, including *Lachnoclostridium, Thermus scotoductus* and *Thermales*. In addition, the study also pointed out that diet, exercise, and sleep may be important correlated factors affecting the changes in the gut microbiota of children with ISS.

However, there are also shortcomings in this study, such as the single-center study does not represent the level of intestinal microbiota in children with ISS in the country, and the retrospective study may have recall bias. Therefore, in the future, we will continue to carry out multi-center, cohort studies and animal experiments to further explore and verify the complexity of the intestinal microbiota of children with ISS, and provide a scientific basis for improving the health and well-being of these children, in order to provide new targets for regulating the intestinal microbiota through probiotics and health science lifestyles, and providing new targets for ISS prevention, etiological treatment and precision medical care.

## Data Availability

The datasets presented in this study can be found in online repositories. The names of the repository/repositories and accession number(s) can be found in the article/supplementary material.

## References

[1] Pedicelli S Peschiaroli E Violi E Cianfarani S. Controversies in the definition and treatment of idiopathic short stature (ISS). J Clin Res In Pediatr Endocrinol. (2009) 1:105–15. doi: 10.4008/jcrpe.v1i3.53, PMID: 21274395 PMC3005647

[2] Guerrini Usubini A Marazzi N Abbruzzese L Tringali G De Micheli R Castelnuovo G. Emotional and behavioural adjustment in children and adolescents with short stature vs. Their normal-statured peers. J Clin Med. (2025) 14:538. doi: 10.3390/jcm14020538, PMID: 39860545 PMC11766412

[3] Bullinger M Kołtowska-Häggström M Sandberg D Chaplin J Wollmann H Noeker M. Health-related quality of life of children and adolescents with growth hormone deficiency or idiopathic short stature - part 2: available results and future directions. Hormone Res. (2009) 72:74–81. doi: 10.1159/000232159, PMID: 19690424

[4] Quitmann JH Bullinger M Sommer R Rohenkohl AC Bernardino Da Silva NM. Associations between psychological problems and quality of life in pediatric short stature from patients’ and parents’ Perspectives. PloS One. (2016) 11:e0153953. doi: 10.1371/journal.pone.0153953, PMID: 27097033 PMC4838264

[5] Ranke MB. Short and long-term effects of growth hormone in children and adolescents with GH deficiency. Front In Endocrinol. (2021) 12:720419. doi: 10.3389/fendo.2021.720419, PMID: 34539573 PMC8440916

[6] Li L Wang Y Huang Y Lu Y Wang W Chen X. Impact of different growth hormone levels on gut microbiota and metabolism in short stature. Pediatr Res. (2024) 96:115–23. doi: 10.1038/s41390-024-03140-4, PMID: 38582946

[7] Yan L Ye B Yang M Shan Y Yan D Fang D. Gut microbiota and metabolic changes in children with idiopathic short stature. BMC Pediatr. (2024) 24:468. doi: 10.1186/s12887-024-04944-3, PMID: 39039462 PMC11265363

[8] Lazar L Eshel A Moadi L Yackobovitch-Gavan M Bar-Maisels M Shtaif B. Children with idiopathic short stature have significantly different gut microbiota than their normal height siblings: a case-control study. Front In Endocrinol. (2024) 15:1343337. doi: 10.3389/fendo.2024.1343337, PMID: 38464968 PMC10920232

[9] Li L Chen L Yang Y Wang J Guo L An J. Characteristics of gut microbiome and its metabolites, short-chain fatty acids, in children with idiopathic short stature. Front In Endocrinol. (2022) 13:890200. doi: 10.3389/fendo.2022.890200, PMID: 35757432 PMC9226366

[10] Cuna A Morowitz MJ Ahmed I Umar S Sampath V. Dynamics of the preterm gut microbiome in health and disease. Am J Physiol Gastrointestinal Liver Physiol. (2021) 320:G411–9. doi: 10.1152/ajpgi.00399.2020, PMID: 33439103 PMC8238167

[11] Jacobson DK Honap TP Ozga AT Meda N Kagoné TS Carabin H. Analysis of global human gut metagenomes shows that metabolic resilience potential for short-chain fatty acid production is strongly influenced by lifestyle. Sci Rep. (2021) 11:1724. doi: 10.1038/s41598-021-81257-w, PMID: 33462272 PMC7813856

[12] Turnbaugh PJ Hamady M Yatsunenko T Cantarel BL Duncan A Ley RE. A core gut microbiome in obese and lean twins. Nature. (2009) 457:480–4. doi: 10.1038/nature07540, PMID: 19043404 PMC2677729

[13] Surono IS Widiyanti D Kusumo PD Venema K. Gut microbiota profile of Indonesian stunted children and children with normal nutritional status. PloS One. (2021) 16:e0245399. doi: 10.1371/journal.pone.0245399, PMID: 33497390 PMC7837488

[14] Hardjo J Selene NB. Stunting and gut microbiota: A literature review. Pediatr Gastroenterology Hepatol Nutr. (2024) 27:137–45. doi: 10.5223/pghn.2024.27.3.137, PMID: 38818278 PMC11134181

[15] Wang X Ye C Xun T Mo L Tong Y Ni W. Bacteroides fragilis polysaccharide A ameliorates abnormal voriconazole metabolism accompanied with the inhibition of TLR4/NF-κB pathway. Front In Pharmacol. (2021) 12:663325. doi: 10.3389/fphar.2021.663325, PMID: 33995087 PMC8115215

[16] Wang X Hu X Ye C Zhao J Tan SC Zhou L. Astragalus Polysaccharide Enhances Voriconazole Metabolism under Inflammatory Conditions through the Gut Microbiota. J Clin Trans Hepatol. (2024) 12:481–95. doi: 10.14218/JCTH.2024.00024, PMID: 38779521 PMC11106349

[17] Zeng Q Feng X Hu Y Su S Luo BR Chen J. Dysbiosis of gut microbiota with enriched pro-inflammatory species in children with idiopathic short stature: a case-control study. Sci Rep. (2025) 15:19779. doi: 10.1038/s41598-025-04569-1, PMID: 40473745 PMC12141481

[18] Rendo-Urteaga T Saravia L Sadalla Collese T Monsalve-Alvarez JM González-Zapata LI Tello F. Reliability and validity of an FFQ for South American children and adolescents from the SAYCARE study. Public Health Nutr. (2020) 23:13–21. doi: 10.1017/S1368980019002064, PMID: 31511116 PMC10200494

[19] Saravia L González-Zapata LI Rendo-Urteaga T Ramos J Collese TS Bove I. Development of a food frequency questionnaire for assessing dietary intake in children and adolescents in south america. Obes (Silver Spring Md.) 26 Suppl. (2018) 1:S31–40. doi: 10.1002/oby.22114, PMID: 29464920

[20] Bull FC Al-Ansari SS Biddle S Borodulin K Buman MP Cardon G. World Health Organization 2020 guidelines on physical activity and sedentary behaviour. Br J Sports Med. (2020) 54:1451–62. doi: 10.1136/bjsports-2020-102955, PMID: 33239350 PMC7719906

[21] Chaput J-P Willumsen J Bull F Chou R Ekelund U Firth J. 2020 WHO guidelines on physical activity and sedentary behaviour for children and adolescents aged 5-17 years: summary of the evidence. Int J Behav Nutr Phys Activity. (2020) 17:141. doi: 10.1186/s12966-020-01037-z, PMID: 33239009 PMC7691077

[22] uysse DJ Reynolds CF Monk TH Berman SR Kupfer DJ. The Pittsburgh Sleep Quality Index: a new instrument for psychiatric practice and research. Psychiatry Res. (1989) 28:193–213. doi: 10.1016/0165-1781(89)90047-4, PMID: 2748771

[23] Birmaher B Brent DA Chiappetta L Bridge J Monga S Baugher M. Psychometric properties of the Screen for Child Anxiety Related Emotional Disorders (SCARED): a replication study. J Am Acad Child Adolesc Psychiatry. (1999) 38:1230–6. doi: 10.1097/00004583-199910000-00011, PMID: 10517055

[24] Yang R Wang Y Ying Z Shi Z Song Y Yan J. Inspecting mother-to-infant microbiota transmission: disturbance of strain inheritance by cesarian section. Front In Microbiol. (2024) 15:1292377. doi: 10.3389/fmicb.2024.1292377, PMID: 38486699 PMC10937581

[25] Berg J Brandt KK Al-Soud WA Holm PE Hansen LH Sørensen SJ. Selection for Cu-tolerant bacterial communities with altered composition, but unaltered richness, via long-term Cu exposure. Appl Environ Microbiol. (2012) 78:7438–46. doi: 10.1128/aem.01071-12, PMID: 22904046 PMC3457098

[26] Michelsen CF Pedas P Glaring MA Schjoerring JK Stougaard P. Bacterial diversity in Greenlandic soils as affected by potato cropping and inorganic versus organic fertilization. Polar Biol. (2014) 37:61–71. doi: 10.1007/s00300-013-1410-9

[27] Magoč T Salzberg SL. FLASH: fast length adjustment of short reads to improve genome assemblies. Bioinf (Oxford England). (2011) 27:2957–63. doi: 10.1093/bioinformatics/btr507, PMID: 21903629 PMC3198573

[28] Bolyen E Rideout JR Dillon MR Bokulich N Abnet CC Al-Ghalith GA. Reproducible, interactive, scalable and extensible microbiome data science using QIIME 2. Nat Biotechnol. (2019) 37:852–7. doi: 10.1038/s41587-019-0209-9, PMID: 31341288 PMC7015180

[29] Kim B-R Shin J Guevarra R Lee JH Kim DW Seol K-H. Deciphering diversity indices for a better understanding of microbial communities. J Microbiol Biotechnol. (2017) 27:2089–93. doi: 10.4014/jmb.1709.09027, PMID: 29032640

[30] Feutrill A Roughan M. A review of shannon and differential entropy rate estimation. Entropy (Basel Switzerland). (2021) 23:1046. doi: 10.3390/e23081046, PMID: 34441186 PMC8392187

[31] Zhang T Domke GM Russell MB Lichstein JW. An index for measuring functional extension and evenness in trait space. Ecol Evol. (2021) 11:7461–73. doi: 10.1002/ece3.7577, PMID: 34188827 PMC8216966

[32] Li MJ Shao DT Zhou JC Gu JH Qin JJ Chen W. Signatures within esophageal microbiota with progression of esophageal squamous cell carcinoma. Chin J Cancer Res. (2020) 32:755–67. doi: 10.21147/j.issn.1000-9604.2020.06.09, PMID: 33446998 PMC7797230

[33] Ronan V Yeasin R Claud EC. Childhood development and the microbiome-the intestinal microbiota in maintenance of health and development of disease during childhood development. Gastroenterology. (2021) 160:495–506. doi: 10.1053/j.gastro.2020.08.065, PMID: 33307032 PMC8714606

[34] Fan Y Pedersen O. Gut microbiota in human metabolic health and disease. Nat Rev Microbiol. (2021) 19:55–71. doi: 10.1038/s41579-020-0433-9, PMID: 32887946

[35] Saghatelyan A Panosyan H Trchounian A Birkeland N-K. Characteristics of DNA polymerase I from an extreme thermophile, Thermus scotoductus strain K1. MicrobiologyOpen. (2021) 10:e1149. doi: 10.1002/mbo3.1149, PMID: 33415847 PMC7884927

[36] De Filippo C Cavalieri D Di Paola M Ramazzotti M Poullet JB Massart S. Impact of diet in shaping gut microbiota revealed by a comparative study in children from Europe and rural Africa. Proc Natl Acad Sci United States America. (2010) 107:14691–6. doi: 10.1073/pnas.1005963107, PMID: 20679230 PMC2930426

[37] David LA Maurice CF Carmody RN Gootenberg DB Button JE Wolfe BE. Diet rapidly and reproducibly alters the human gut microbiome. Nature. (2014) 505:559–63. doi: 10.1038/nature12820, PMID: 24336217 PMC3957428

[38] Walker AW Ince J Duncan SH Webster LM Holtrop G Ze X. Dominant and diet-responsive groups of bacteria within the human colonic microbiota. ISME J. (2011) 5:220–30. doi: 10.1038/ismej.2010.118, PMID: 20686513 PMC3105703

[39] Singh RK Chang H-W Yan D Lee KM Ucmak D Wong K. Influence of diet on the gut microbiome and implications for human health. J Trans Med. (2017) 15:73. doi: 10.1186/s12967-017-1175-y, PMID: 28388917 PMC5385025

[40] Etxeberria U Fernández-Quintela A Milagro FI Aguirre L Martínez JA Portillo MP. Impact of polyphenols and polyphenol-rich dietary sources on gut microbiota composition. J Agric Food Chem. (2013) 61:9517–33. doi: 10.1021/jf402506c, PMID: 24033291

[41] Ross FC Patangia D Grimaud G Lavelle A Dempsey EM Ross RP. The interplay between diet and the gut microbiome: implications for health and disease. Nat Rev Microbiol. (2024) 22:671–86. doi: 10.1038/s41579-024-01068-4, PMID: 39009882

[42] De Filippis F Pellegrini N Vannini L Jeffery IB La Storia A Laghi L. High-level adherence to a Mediterranean diet beneficially impacts the gut microbiota and associated metabolome. Gut. (2016) 65:1812–21. doi: 10.1136/gutjnl-2015-309957, PMID: 26416813

[43] Cheng B Feng H Li C Jia F Zhang X. The mutual effect of dietary fiber and polyphenol on gut microbiota: Implications for the metabolic and microbial modulation and associated health benefits. Carbohydr Polymers. (2025) 358:123541. doi: 10.1016/j.carbpol.2025.123541, PMID: 40383597

[44] Pan L Farouk MH Qin G Zhao Y Bao N. The influences of soybean agglutinin and functional oligosaccharides on the intestinal tract of monogastric animals. Int J Mol Sci. (2018) 19:554. doi: 10.3390/ijms19020554, PMID: 29439523 PMC5855776

[45] Menni C Jackson MA Pallister T Steves CJ Spector TD Valdes AM. Gut microbiome diversity and high-fibre intake are related to lower long-term weight gain. Int J Obes (2005). (2017) 41:1099–105. doi: 10.1038/ijo.2017.66, PMID: 28286339 PMC5500185

[46] Wu GD Chen J Hoffmann C Bittinger K Chen Y-Y Keilbaugh SA. Linking long-term dietary patterns with gut microbial enterotypes. Sci (New York N.Y.). (2011) 334:105–8. doi: 10.1126/science.1208344, PMID: 21885731 PMC3368382

[47] Ley RE Turnbaugh PJ Klein S Gordon JI. Microbial ecology: human gut microbes associated with obesity. Nature. (2006) 444:1022–3. doi: 10.1038/4441022a, PMID: 17183309

[48] Rinninella E Raoul P Cintoni M Franceschi F Miggiano GAD Gasbarrini A. What is the healthy gut microbiota composition? A changing ecosystem across age, environment, diet, and diseases. Microorganisms. (2019) 7:14. doi: 10.3390/microorganisms7010014, PMID: 30634578 PMC6351938

[49] Sonnenburg ED Smits SA Tikhonov M Higginbottom SK Wingreen NS Sonnenburg JL. Diet-induced extinctions in the gut microbiota compound over generations. Nature. (2016) 529:212–5. doi: 10.1038/nature16504, PMID: 26762459 PMC4850918

[50] Estaki M Pither J Baumeister P Little JP Gill SK Ghosh S. Cardiorespiratory fitness as a predictor of intestinal microbial diversity and distinct metagenomic functions. Microbiome. (2016) 4:42. doi: 10.1186/s40168-016-0189-7, PMID: 27502158 PMC4976518

[51] Mohr AE Jäger R Carpenter KC Kerksick CM Purpura M Townsend JR. The athletic gut microbiota. J Int Soc Sports Nutr. (2020) 17:24. doi: 10.1186/s12970-020-00353-w, PMID: 32398103 PMC7218537

[52] Miquel S Martín R Rossi O Bermúdez-Humarán LG Chatel JM Sokol H. Faecalibacterium prausnitzii and human intestinal health. Curr Opin In Microbiol. (2013) 16:255–61. doi: 10.1016/j.mib.2013.06.003, PMID: 23831042

[53] Clarke SF Murphy EF O’Sullivan O Lucey AJ Humphreys M Hogan A. Exercise and associated dietary extremes impact on gut microbial diversity. Gut. (2014) 63:1913–20. doi: 10.1136/gutjnl-2013-306541, PMID: 25021423

[54] Clauss M Gérard P Mosca A Leclerc M. Interplay between exercise and gut microbiome in the context of human health and performance. Front In Nutr. (2021) 8:637010. doi: 10.3389/fnut.2021.637010, PMID: 34179053 PMC8222532

[55] Smith RP Easson C Lyle SM Kapoor R Donnelly CP Davidson EJ. Gut microbiome diversity is associated with sleep physiology in humans. PloS One. (2019) 14:e0222394. doi: 10.1371/journal.pone.0222394, PMID: 31589627 PMC6779243

[56] Benedict C Vogel H Jonas W Woting A Blaut M Schürmann A. Gut microbiota and glucometabolic alterations in response to recurrent partial sleep deprivation in normal-weight young individuals. Mol Metab. (2016) 5:1175–86. doi: 10.1016/j.molmet.2016.10.003, PMID: 27900260 PMC5123208

[57] O’Mahony SM Clarke G Borre YE Dinan TG Cryan JF. Serotonin, tryptophan metabolism and the brain-gut-microbiome axis. Behav Brain Res. (2015) 277:32–48. doi: 10.1016/j.bbr.2014.07.027, PMID: 25078296

[58] Khan MT Zohair M Khan A Kashif A Mumtaz S Muskan F. From Gut to Brain: The roles of intestinal microbiota, immune system, and hormones in intestinal physiology and gut-brain-axis. Mol Cell Endocrinol. (2025) 607:112599. doi: 10.1016/j.mce.2025.112599, PMID: 40482955

[59] Matenchuk BA Mandhane PJ Kozyrskyj AL. Sleep, circadian rhythm, and gut microbiota. Sleep Med Rev. (2020) 53:101340. doi: 10.1016/j.smrv.2020.101340, PMID: 32668369

[60] Shimizu Y Yamamura R Yokoi Y Ayabe T Ukawa S Nakamura K. Shorter sleep time relates to lower human defensin 5 secretion and compositional disturbance of the intestinal microbiota accompanied by decreased short-chain fatty acid production. Gut Microbes. (2023) 15:2190306. doi: 10.1080/19490976.2023.2190306, PMID: 36945116 PMC10038026

[61] Ticinesi A Lauretani F Tana C Nouvenne A Ridolo E Meschi T. Exercise and immune system as modulators of intestinal microbiome: implications for the gut-muscle axis hypothesis. Exercise Immunol Rev. (2019) 25:84–95., PMID: 30753131

[62] Mansolf M Blackwell CK Cummings P Choi S Cella D. Linking the child behavior checklist to the strengths and difficulties questionnaire. psychol Assess. (2022) 34:233–46. doi: 10.1037/pas0001083, PMID: 34843282 PMC9718585

[63] McDonnell L Gilkes A Ashworth M Rowland V Harries TH Armstrong D. Association between antibiotics and gut microbiome dysbiosis in children: systematic review and meta-analysis. Gut Microbes. (2021) 13:1–18. doi: 10.1080/19490976.2020.1870402, PMID: 33651651 PMC7928022

